# Real-world comparison of the efficacy of first-line therapies and the influence of risk factors in advanced renal cell carcinoma

**DOI:** 10.1007/s12672-025-02131-z

**Published:** 2025-03-19

**Authors:** Maximilian Haack, Stephanie Neuberger, Jan Hendrik Boerner, Stefanie Ziewers, Gregor Duwe, Robert Dotzauer, Axel Haferkamp, Rene Mager

**Affiliations:** Department of Urology and Pediatric Urology, Johannes Gutenberg University Medical Center, Langenbeckstraße 1, 55131 Mainz, Rhineland-Palatinate Germany

**Keywords:** Advanced renal cell carcinoma, Immunotherapy, Targeted therapy, Real-world data

## Abstract

**Introduction:**

Systemic therapy for advanced renal cell carcinoma (aRCC) has become increasingly diverse. In the 1st-line setting, various combination therapies are available, with little comparative data on the efficacy of the therapies. The aim of this study was to compare the current 1st-line combination therapies under real-life conditions and to investigate risk factors in the patient population.

**Methods:**

Patients with aRCC who started 1st-line IO/IO or IO/TKI combination  therapy between 03/2019 and 10/2023 were included. The primary endpoints were progression-free survival (PFS) and overall survival (OS). Secondary endpoints were time on treatment (ToT), duration of response (DoR), subsequent therapies, the evaluation of risk factors and their influence on PFS and OS. Survival data were analysed using Kaplan–Meier estimates with log-rank tests, risk factors for PFS and OS using Cox regression analysis.

**Results:**

A total of 59 patients, mainly men (79.7%) with a median age of 64.8 years were included. The median follow-up was 21 months. The comparison of IO/IO vs. IO/TKI demonstrated a median PFS of 6 (2.08–9.92) vs. 14 (9.06–18.94) months (47 events; HR IO/TKI vs. IO/IO: 0.53 (0.29–0.99); p = 0.039) and a median OS of 20 (15.07–24.94) vs. 33 (21.68–44.32) months (32 deaths; HR IO/TKI vs. IO/IO: 0.74 (0.36–1.51); p = 0.403). Off all risk factors analysed only synchronous metastases proved to be of independent predictive value for PFS (HR 2.38; 95% CI 1.11–5.11; p = 0.026) and OS (HR 3.47; 95% CI 1.15–10.44; p = 0.027).

**Conclusion:**

An IO/TKI therapy showed a significantly improved PFS in the real-world setting compared to an IO/IO combination. In terms of OS, the improved treatment response of the IO/TKI group did not prevail.

**Supplementary Information:**

The online version contains supplementary material available at 10.1007/s12672-025-02131-z.

## Introduction

Renal cell carcinoma (RCC) accounts for around 2–3% of all malignant diseases in adults and is responsible for about 180,000 deaths per year worldwide [1]. The prognosis of advanced renal cell carcinoma (aRCC) remains poor, with a five-year survival rate of less than 15% despite advanced treatment strategies [1]. Historically, monotherapies such as cytokines and later tyrosine kinase inhibitors (TKIs) were the mainstay of treatment for aRCC. However, these approaches have proven to be of limited efficacy when it comes to controlling the disease in the long term and ensuring survival.

In recent years, the therapeutic landscape for aRCC has evolved considerably with the emergence of combination therapies [2]. These therapies usually combine immune-oncologicals (IO) with TKIs or two IOs together. Clinical trials have shown superior efficacy of combination therapies compared to monotherapy approaches [3–8]. Such combinations aim to exploit the synergistic effects of influencing multiple metabolic and immunogenic pathways involved in tumor growth. Where the combination of two IOs exclusively focusses on the T-cell interaction with the tumor cells, the combination of an IO with a TKI additionally targets mechanisms of proliferation and angiogenesis. Herein lies the fundamental difference between the two therapy modalities. Although recent follow-up data from the CheckMate 214 trial has shown impressive long-term survival in patients treated with Ipilimumab/Nivolumab, this only applies to a subset of responders [9]. In addition, this is of limited use in decision making when compared to the various IO/TKI regimens in first-line treatment of aRCC, which also show promising long-term efficacy [10–13].

Due to the large variety of TKIs, far more therapy combinations of IO with TKIs are available. However, since the efficacy of the individual therapy combinations is usually tested against a TKI monotherapy (usually against sunitinib), there is still too little evidence about the comparative efficacy of the therapy combinations with each other. This general paucity of studies is compounded by the lack of real-world data outside the North American continent. Among other things, differences in living conditions, healthcare systems and treatment practices make it difficult to compare these data in an international context. Few recent studies from Europe provide comparable data in this respect [14–16]. To address these issues, we sought to enhance the limited data landscape by further comparing the efficacy of current first-line combination therapies for aRCC under real-world conditions in our large tertiary centre in Germany. In this context, we focused on the efficacy of the two fundamental treatment strategies IO/IO vs. IO/TKI with regard to progression free survival (PFS), duration of response (DoR), time on treatment (ToT) and overall survival (OS). Furthermore, we analyzed the influence of potential risk factors in the patient population as well as comparing the side effect profile, treatment effort and adherence of the individual treatment combinations to each other.

## Materials and methods

### Study design and population

**Fig. 1:**
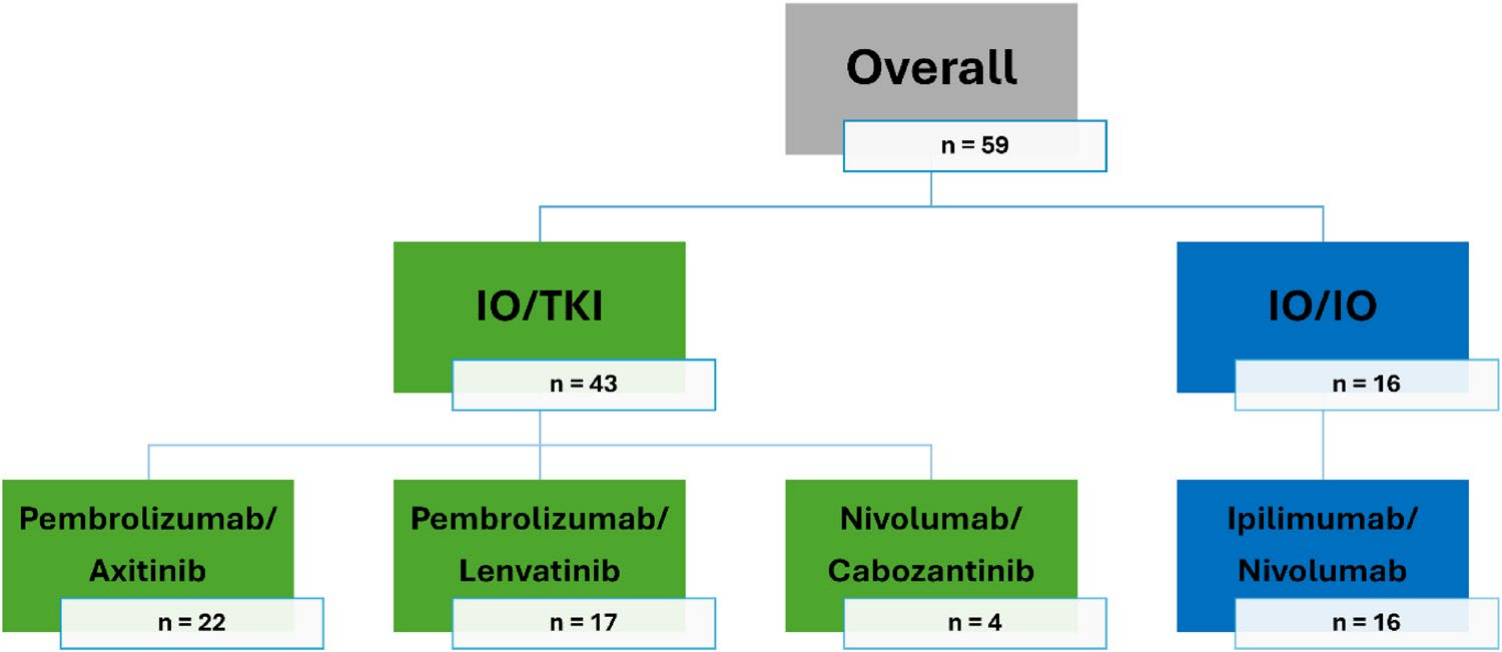
Distribution of the study population into the corresponding combination therapies of immuno-oncologicals (IO) and tyrosine kinase inhibitors (TKI)

In this single center, observational study, we retrospectively investigated patients with aRCC, who received one of the first line combination therapies listed below at the Department of Urology and Paediatric Urology at the University Hospital Mainz, Germany (Fig. 1). The study protocol was reviewed and approved by the ethics committee of the state medical association of Rhineland-Palatinate, approval number 2024-17599-retrospective. Data processing was carried out in accordance with the applicable guidelines of the Rhineland-Palatinate State Hospital Act (§36, 37).

Therapeutic regimes were in accordance with the corresponding authorisation studies:

- Pembrolizumab (200 mg, intravenously) once every 3 weeks and Axitinib (5 mg, orally) twice daily [3]
- Pembrolizumab (200 mg, intravenously) once every 3 weeks and Lenvatinib (20 mg, orally) once daily [7]
- Nivolumab (240 mg, intravenously) once every 2 weeks and Cabozantinib (40 mg, orally) once daily [8]
- Ipilimumab (1 mg per kilogram, intravenously) and Nivolumab (3 mg per kilogram, intravenously) combined once every 3 weeks for four doses (induction phase), followed by Nivolumab monotherapy (3 mg per kilogram, intravenously) once every 2 weeks (maintenance phase) [5]

Primary outcomes were PFS and OS. Disease progression was assessed every 3 months by experienced radiologists at our centre as part of routine staging examinations. The Response Evaluation Criteria in Solid Tumours (RECIST) were strictly adhered to as standard. Secondary outcomes were time on treatment (ToT), duration of response (DoR), subsequent therapies, PFS of second line treatment, basic patient characteristics, comorbidities and oncological parameters (histology, IMDC score, cytoreductive surgery, distribution, number and timing of metastases) as well as side effect profile (type and severity according to the Common Terminology Criteria for Adverse Events (CTCAE)), treatment effort and adherence (number of interdisciplinary consultations, dose reduction and use of corticosteroids). The time of metastasis was divided into synchronous (< 3 months after diagnosis) and metachronous (> 3 months after diagnosis).

Oncological outcomes were defined as follows:

- PFS: The time from treatment initiation to disease progression or death from any cause.
- OS: The time from treatment initiation to death from any cause.
- DoR: The time from the first documented response (partial or complete response according to RECIST) to disease progression or death from any cause [17].
- ToT: The time from treatment initiation to the date of the last administered dose.

### Statistical analysis

Kaplan-Maier estimates with Peto-Pike’s Chi^2^ as log-rank test were performed to analyse primary endpoints (PFS and OS). Hazard ratios with 95% confidence intervals (CI) were approximated via Peto-Pike. Continuous variables were tested by Mann Whitney U test. Categorical variables were tested using Chi square or Fisher’s exact test. Univariate and multivariate cox-regression analysis were performed to identify independent predictors for primary endpoints. The level of significance was set at p < 0.05.

## Results

### Basic patient characteristics

Overall, a total of 59 patients were included from March 2019 to October 2023. The majority of patients were male (79.7%) with a median age of 68 (IQR 58–76) in IO/TKI group and 59 (IQR 51–66) in IO/IO group (p = 0.06). Patients in IO/IO group significantly more often had an Eastern Cooperative Oncology Group status (ECOG) of 1 (81%) compared to IO/TKI (42%) but had no ECOG status of ≥ 2 compared to IO/TKI (7%) (p = 0.02). Comorbidities recorded in the study population were indifferent between IO/TKI and IO/IO (Table 1).

**Table 1:** Basic patient characteristics of the study population

Variable	IO/IO N = 16	IO/TKI N = 43	p-value
Age, [a], median (IQR)	59 (51-66)	68 (58-76)	0.06
Male/Female, n	10/6	37/6	0.06
BMI, [kg*m^2^], median (IQR)	28 (23-31)	27 (24-30)	0.7
ECOG, n (%)			
0	3 (19)	22 (51)	0.02
1	13 (81)	18 (42)	
≥ 2	0 (0)	3 (7)	
ASA, n (%)			
1–2	11 (69)	25 (58)	0.7
3–4	5 (31)	18 (42)	
Comorbidities, n (%)			
History of smoking	4 (25)	19 (44)	0.3
Pack years, median (IQR)	23 (13-34)	25 (15-30)	0.9
Arterial hypertension	11 (69)	22 (51)	0.4
Chronic heart failure	0 (0)	1 (0)	1.0
Atrial fibrillation	1 (1)	7 (16)	0.6
Coronary artery disease	0 (0)	3 (1)	0.6
Peripheral artery disease	0 (0)	1 (0)	1.0
Anemia	1 (1)	6 (14)	0.7
Liver cirrhosis	0 (0)	3 (1)	0.6
Diabetes mellitus	4 (25)	8 (19)	0.7
Chronic kidney disease	0 (0)	8 (19)	0.1
COPD	1 (1)	7 (16)	0.6
2nd cancerous disease	1 (1)	10 (23)	0.3
Thyroid disease	0 (0)	3 (1)	0.6
Colitis	1 (1)	0 (0)	0.3
Neurologic disorder	1 (1)	5 (12)	1.0

Continuous variables are presented as median and interquartile range and were tested by Mann Whitney U test. Categorical variables were tested using Chi square or Fisher’s exact test. P < 0.05 was considered significant

### Oncological parameters

There was no difference in histology between the treatment groups, with approximately 80% of cases being of the clear cell subtype (Table 2). In addition, just over half of the patients in both groups equally underwent cytoreductive surgery (Table 2). Regarding the distribution of risk groups according to International mRCC Database Consortium (IMDC), there were no significant differences for the intermediate and poor risk profiles (Table 2). Due to the authorisation, naturally only patients in the IO/TKI group had a low risk profile according to IMDC (Table 2). In terms of number, distribution and timepoint of metastasis there was also no significant difference between the two therapeutic regimes (Table 2). However, only patients in the IO/TKI group (13%) showed cerebral metastases (Table 2). A comparison of the two treatment combinations IO/IO and IO/TKI showed no significant differences in terms of IMDC score, number of metastases and distribution (Table 2).

**Table 2:** Oncological parameters of the study population

Variable	IO/IO N = 16	IO/TKI N = 43	p-value
Histology			
Clear cell	13 (81)	35 (83)	1.0
Non-clear cell	3 (19)	7 (17)	
IMDC, n (%)			
Good	0 (0)	8 (19)	0.1
Intermediate	12 (75)	22 (51)	0.2
Poor	4 (25)	13 (30)	0.8
Cytoreductive surgery			
No	7 (44)	20 (47)	1.0
Yes	9 (56)	23 (53)	
Synchronous metastases, n (%)	12 (75)	25 (58)	0.4
Distribution of metastases, n (%)			
Pulmonary	11 (69)	29 (67)	1.0
Visceral	6 (38)	20 (47)	0.7
Cerebral	0 (0)	5 (12)	0.3
Osseous	3 (19)	13 (30)	0.5
Lymphnodes	9 (56)	19 (44)	0.6

Continuous variables are presented as median and interquartile range and were tested by Mann Whitney U test. Categorical variables were tested using Chi square or Fisher’s exact test. P < 0.05 was considered significant. IMDC: International mRCC Database Consortium

### Side effects, treatment effort and adherence

A comparison of the two treatment regimens (IO/IO vs. IO/TKI) showed a largely similar occurrence of treatment related adverse events (TRAE) defined after CTCAE (median (IQR); 4.5 (3–5.8) vs. 5.0 (3.8–6), p = 0.5) (Table 3). In both groups, a TRAE ≥ 3 was rare (median (IQR); 1 (1–2) vs. 1 (1–2), p = 0.9). Interestingly, of the worst TRAE, life-threatening side effects (CTCAE grade 4) occurred only in the IO/TKI group (19%, p = 0.3). No patient died due to TRAE. Treatment related drug discontinuation (TRDD) was insignificantly more common in IO/TKI (14%) compared to IO/IO (6%) (p = 0.7; Table 3). The use of glucocorticoid therapy was similarly distributed in both groups (33% vs. 23%, p = 0.5) (Table 3). A dose reduction only occurred in the IO/TKI group due to the possibility of dose reduction when using TKIs. The number of specialist consultations for multidisciplinary management of adverse effects was significantly higher in the IO/TKI group than in the IO/IO group. (median (IQR); 2 (1–4) vs. 0,5 (0–2.75); p = 0.03).

**Table 3:** Side effect profile (type and severity according to Common Terminology Criteria for Adverse Events (CTCAE)), treatment effort and adherence (number of interdisciplinary consultations, dose reduction and use of corticosteroids)

Variable	IO/IO N = 16	IO/TKI N = 43	p-value
TRAE, median (IQR)	4.5 (3–5.8)	5 (3.8–6)	0.5
TRAE ≥ grade 3, median (IQR)	1 (1–2)	1 (1–2)	0.9
Worst TRAE grade, n (%)			
1	4 (25)	8 (19)	0.3
2	6 (38)	10 (23)	
3	6 (38)	16 (37)	
4	0 (0)	8 (19)	
TRDD, n (%)	1 (6)	6 (14)	0.7
Glucorticoid therapy, n (%)	5 (33)	10 (23)	0.5
Dose reduction, n (%)	0 (0)	23 (54)	
Speciality consultation, median (IQR)	0.5 (0–2.75)	2 (1–4)	0.03

Continuous variables are presented as median and interquartile range and were tested by Mann Whitney U test. Categorical variables were tested using Chi square or Fisher’s exact test. P < 0.05 was considered significant. TRAE: treatment related adverse event; TRDD: treatment related drug discontinuation

### Survival outcomes and response dynamics

**Fig. 2:**
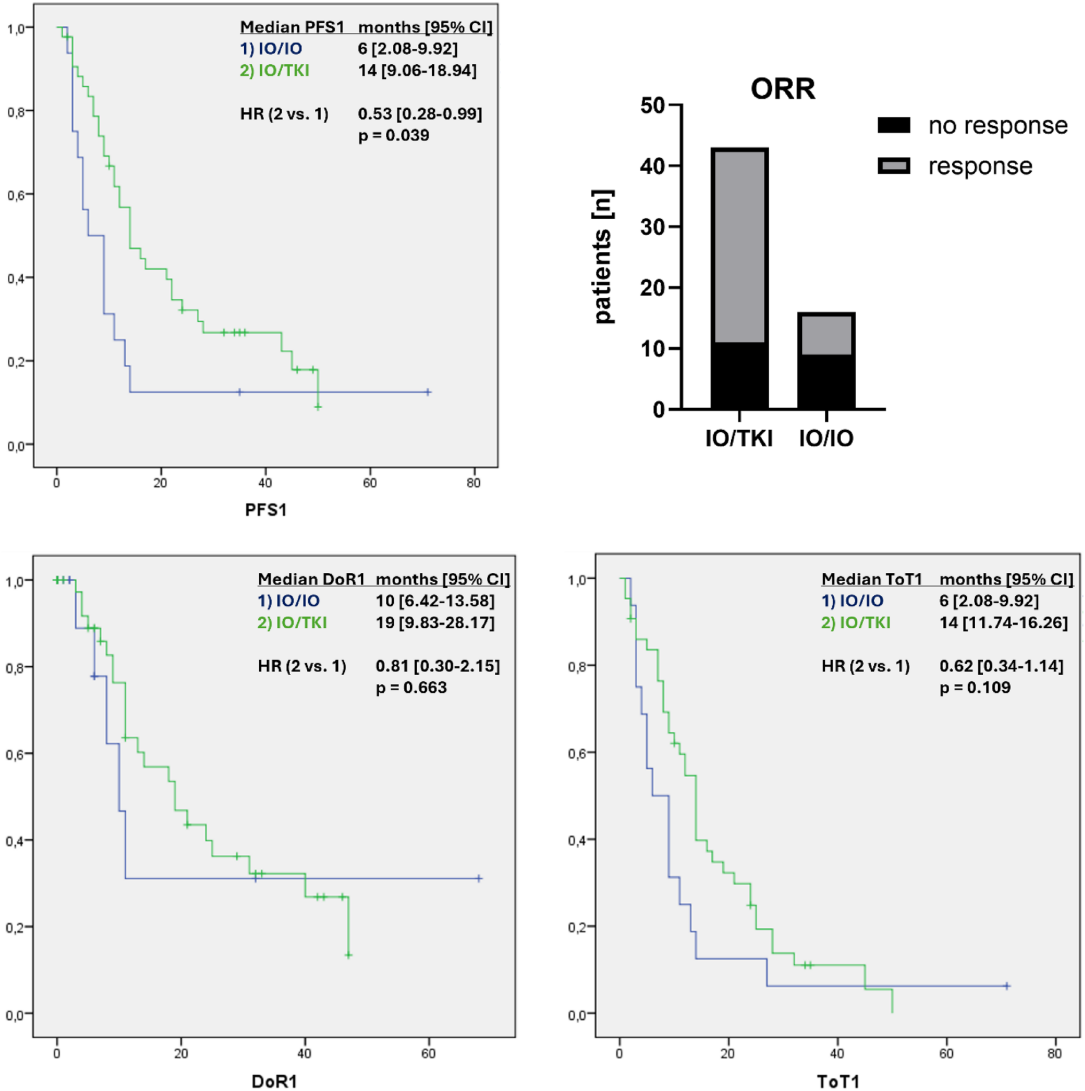
Kaplan-Maier analysis of progression-free survival (PFS1), duration of response (DoR1) and time on treatment (ToT1) as well as objective response rate (ORR) of the first line treatment compared between the two therapy regimes (1. IO/IO and 2. IO/TKI). Peto-Pike’s Chi^2^ was used for Log-Rank-Test. Hazard ratios (HR) with 95% confidence intervals (CI) were approximated via Peto-Pike. P < 0.05 was considered significant

Median follow up was 21 months in total, 19 month (IQR 9–33) in IO/IO and 22 month (IQR 11–38) in IO/TKI (p = 0.816) (supplementary Fig. 5). When analysing the two treatment groups IO/IO vs. IO/TKI regarding oncological outcome data in the first line setting, the IO/TKI group showed significantly better PFS1 with 6 (2.08–9.92) vs. 14 (9.06–18.94) months (14 and 33 events; HR IO/TKI vs. IO/IO: 0.53 (0.28–0.99); p = 0.039) (Fig. 2) and an improved objective response rate (ORR) (7/16 (44%) vs. 32/43 (74%), p = 0.035) (Fig. 2). However, the median DoR1, with 10 (6.42–13.58) vs. 19 (9.83–28.17) months (5 and 23 events), did not differ significantly (HR IO/TKI vs. IO/IO 0.81 (0.30–2.15); p = 0.663) (Fig. 2). At 6 (2.08–9.92) vs. 14 (11.74–16.26) months (15 and 38 events; HR IO/TKI vs. IO/IO 0.62 (0.34–1.14); p = 0.109), median ToT1 was almost identical to PFS1, as 71% of discontinuations were due to progression leading to second-line treatment. Treatment was discontinued in only 7 patients due to side effects (Table 3) and in 5 patients due to a durable response. There was still a significant improvement in PFS in the IO/TKI group when analysing only intermediate and poor risk patients (6 (2.08–9.92) vs. 14 (8.50–19.50) months; 14 and 26 events; HR IO/TKI vs. IO/IO 0,52 (0.27–0.99); p = 0.042) (supplementary Fig. 1).

**Fig. 3:**
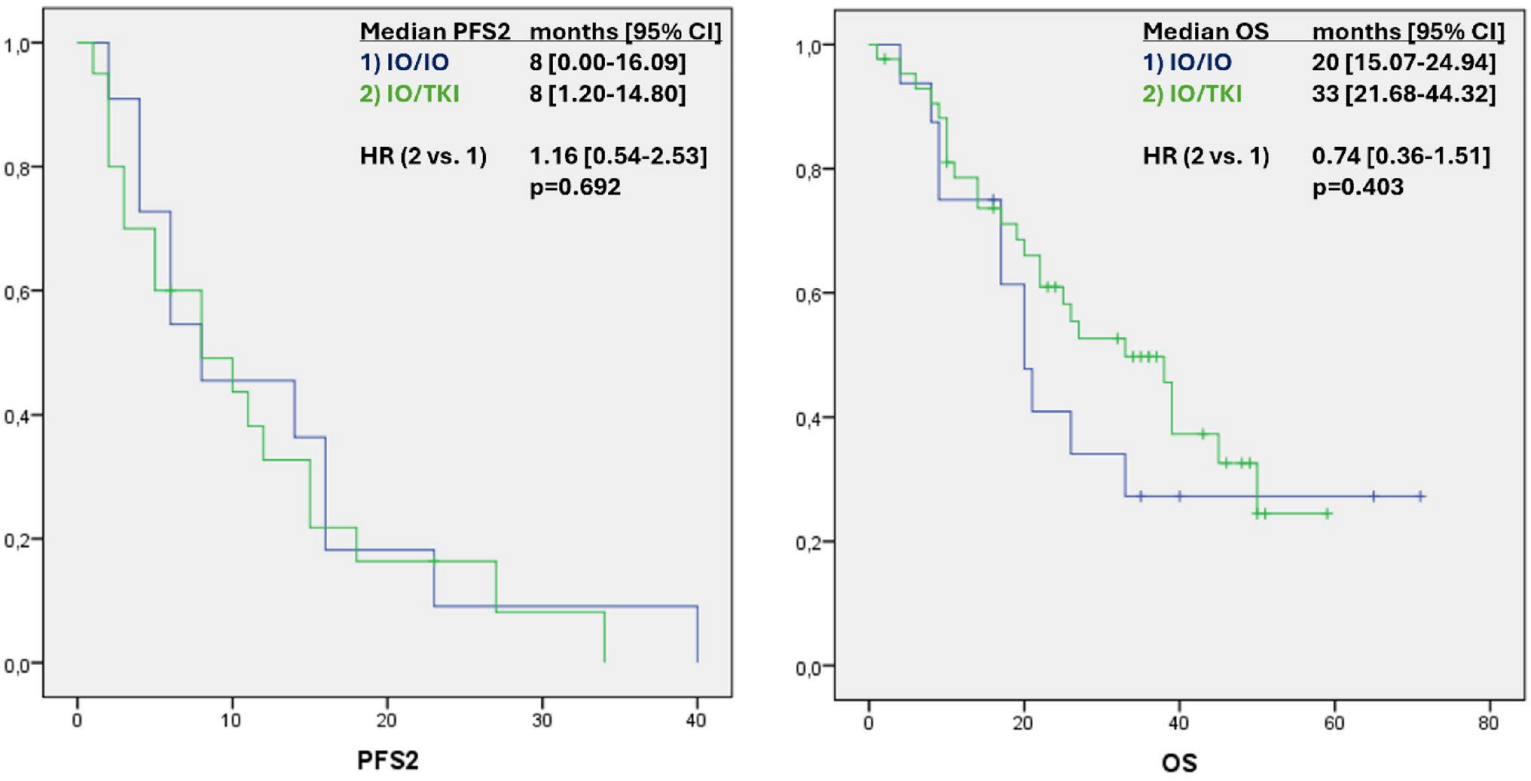
Kaplan-Maier analysis of progressive free survival of second line treatment (PFS2) and overall survival (OS) compared between the two therapy regimes (1. IO/IO and 2. IO/TKI). Peto-Pike’s Chi^2^ was used for Log-Rank-Test. Hazard ratios (HR) with 95% confidence intervals (CI) were approximated via Peto-Pike. P < 0.05 was considered significant

In the second line setting, the majority of patients received TKI monotherapy (72%) (supplementary Fig. 4). The next most common second-line therapy was the combination of Lenvatinib/Everolimus (22%) (supplementary Fig. 4). Due to the vast majority of second line TKI monotherapies in both initial treatment arms, there was no difference in PFS2 between the two groups (8 (0–16.09) vs. 8 (1.20–14.80) months; 11 and 18 events; HR IO/TKI vs. IO/IO 1,16 (0.54–2.53); p = 0.692) (Fig. 3). In terms of OS, the improved treatment response of the IO/TKI group compared to the IO/IO group did not prevail, although there was a visible difference (20 (15.07–24.94) vs. 33 (21.68–44.32) months; 11 and 25 deaths; HR IO/TKI vs. IO/IO 0.74 (0.36–1.51); p = 0.403) (Fig. 3). When analysing only intermediate and poor risk patients, there was also no significant difference regarding OS (20 (15.07–24.94) vs. 38 (24–52.01) months; 11 and 21 deaths; HR IO/TKI vs. IO/IO 0.79 (0.38–1.63); p = 0.510) (supplementary Fig. 2).

Due to the small study population, there was no significant difference between the individual treatment combinations within the IO/TKI group or compared to the IO/IO group for PFS and OS (Supplementary Fig. 3).

### Risk factor analysis

When examining basic patient characteristics and comorbidities no risk factors for the oncological outcome parameters were identified. In comparison to IO/IO univariate Cox regression analysis showed a positive correlation between PFS and treatment with IO/TKI (HR 0.46; 95% CI 0.23–0.90; p = 0.023) (supplementary Table 1). Multivariate Cox regression analysis confirmed IO/TKI combination as an independent predictor of improved PFS (HR 0.47; 95% CI 0.24–0.92; p = 0.029) (supplementary Table 3). Interestingly, IO/TKI treatment remained an independent predictor of PFS in multivariate Cox regression analysis even after good risk patients were excluded (HR 0.44; 95% CI 0.22–0.89; p = 0.022) (supplementary Table 3). With respect to OS, the Cox regression analysis already indicated a positive correlation between the combination of IO/TKI and OS (supplementary Table 2). However, consistent with the Kaplan–Meier analysis (Fig. 3), this was not significant (HR 0.62; 95% CI 0.26–1.49; p = 0.283) (supplementary Table 4).

Regarding time to progression, univariate Cox regression analysis showed a negative correlation between synchronous metastases and PFS (HR 2.42; 95% CI 1.13–5.15; p = 0.022), but no correlation between PFS and the individual distribution of metastases (supplementary Table 1). Multivariate Cox regression analysis confirmed the occurrence of synchronous metastases as an independent predictor of poorer PFS (HR 2.38; 95% CI 1.11–5.11; p = 0.026) (supplementary Table 3). In line with this, there was also a negative correlation between synchronous metastasis and OS (HR 3.79; 95% CI 1.28–11.27; p = 0.016) (supplementary Table 2), which emerged as the most significant predictor of poorer OS in multivariate Cox regression (HR 3.47; 95% CI 1.15–10.44; p = 0.027) (supplementary Table 4). The most favourable metastatic pattern was seen in pulmonary metastases alone (HR 0.42; 95% CI 0.18–0.97; p = 0.043) (supplementary Table 2). However, this correlation did not prove to be an independent predictor of poorer OS in multivariate Cox regression analysis (HR 0.47; 95% CI 0.20–1.10; p = 0.082) (supplementary Table 4).

## Discussion

Due to the rapid development of 1st-line therapies for aRCC, several treatment combinations are now available. Little evidence about the comparative efficacy of these combination therapies makes a differentiated and well-founded selection difficult. In our study, we aimed to compare the real-world efficacy of different first-line therapies for aRCC at our uro-oncological center. As a fundamental difference in treatment strategies, we investigated the efficacy of the combinations of two IOs (Ipilimumab + Nivolumab) with the combination of an IO and TKI (Pembrolizumab + Axitinib; Pembrolizumab + Lenvatinib; Nivolumab + Cabozantinib).

We were able to show a significantly improved PFS and ORR with the combination of IO/TKI (Fig. 2). Our data suggest a superior efficacy of IO/TKI, which has been shown before in a few comparative studies [15, 18–21]. Regarding OS, we also observed a visible improvement in the IO/TKI group, which however was not significant (Fig. 3). Unsurprisingly for real-world data, we could not achieve similar PFS and OS in the treatment groups, compared to the corresponding pivotal studies [3, 5, 7, 8]. The more frequent response rate and improved PFS of IO/TKI is probably due to the different therapeutic targets on immunogenic activation and inhibition of proliferation and angiogenesis, rather than exclusively targeting T-cell interaction with an IO/IO combination [19–21]. Although overall responses to IO/IO therapy were less frequent, a small number of patients showed remarkable long-term survival. This observation is consistent with the long-term data from CheckMate 214 [9], which continues to make this combination of treatments so attractive for young patients with low tumor burden.

Since the treatment combination IO/IO is only approved for intermediate/poor risk patients according to IMDC [5], it is reasonable to assume that the superior efficacy of IO/TKI on PFS shown here is based on a large proportion of good risk patients. However, at only 19% this proportion is low in our study population (Table 2). In addition, the other two risk groups are balanced for both treatment strategies (intermediate: 75% vs. 51%, p = 0.2; poor: 25% vs. 30%, p = 0.8) (Table 2). When good risk patients were excluded from the analysis, leaving only intermediate and poor risk patients, the IO/TKI group still showed a significantly improved PFS compared to IO/IO, highlighting the universal applicability of this combination therapy and may help guide treatment decisions. Time on treatment and duration of response are becoming increasingly important as additional outcome parameters in current clinical trials to further differentiate treatment efficacy [17]. The DoR in our patient population did not confirm the improved therapeutic effect of the IO/TKI group as seen in PFS, which probably was due to the lower number of patients meeting the DoR criteria. As only 7 patients in the IO/IO group showed any objective response at all and two of them still did not reach the DoR endpoint (progression or death), only 5 patients remained for statistical analysis. In the IO/TKI group, 23 patients still achieved DoR. This may also explain why median DoR was longer than PFS in both groups. In our small patient population, comparisons of DoR are therefore limited. The fact that the rate of non-responders (19% vs. 14%) and patients with insufficient response (38% vs. 14%) was higher in the IO/IO group should not be ignored. Since the most common reason for a change in treatment was tumor progression (71%), ToT was almost identical to PFS. In seven patients treatment was stopped due to side effects and in five patients because they achieved a long-lasting treatment response. This underlines the overall good tolerability of both regimens. As the second-line follow-up therapies were largely TKI monotherapies, an equal PFS between the groups was not surprising. Therefore, the observed (although not significant) difference in OS could be due to the improved efficacy of the IO/TKI combination in the first line setting. The data from our relatively small patient population are therefore consistent with other recent real-world studies that have also shown improved efficacy of an IO/TKI combination in terms of oncological outcomes. [14, 16, 22, 23]. In particular, the study by Yanagisawa et al. supports our findings with a large, multicentric patient population and an IMDC-based propensity score matched analysis conducted in Japan [23].

When looking at number, time and distribution of metastases, we showed that metastatic spreading was broader but not more frequent in the IO/TKI group compared to IO/IO (Table 2). In particular, cerebral metastases occurred exclusively in the IO/TKI group, which are known to be responsible for a significantly poorer survival prognosis [24, 25]. Nevertheless, we could not identify a metastatic distribution that was associated with significantly worse PFS or OS (supplementary Table 1). Only pulmonary metastasis alone was the most favourable metastatic pattern with the lowest risk of poorer PFS and OS, even though this is considered to be the most common site of metastasis [26–28]. Our data thus confirm the findings of Wei et al., who also demonstrated a more favourable prognosis for lung metastases alone compared to other metastatic sites [27]. Their retrospective analysis of the Surveillance, Epidemiology, and End Results (SEER) database of 10,410 patients showed that OS was approximately twice as long in patients with only pulmonary or bone metastases compared with those with only hepatic or cerebral metastases [27]. However, another retrospective analysis of 1151 patients with propensity score matching by Kang et al. showed that the presence of osseous metastases was an independent predictor of poorer OS compared to no osseous metastases [29]. Pulmonary metastases therefore appear to have a more favourable prognosis for overall survival. However, further studies are needed that specifically address the risk distribution of different metastatic patterns in aRCC in relation to PFS and OS. In terms of time to progression, we were able to show that synchronous metastasis led to significantly worse PFS and OS. In fact, it was the strongest independent risk factor overall (supplementary Table 3 and 4). These results are consistent with other studies that have also identified synchronous metastases as an independent risk factor for PFS and OS [28, 30, 31].

In terms of comorbidities and basic patient characteristics, the median age of patients in the IO/TKI cohort was noticeably older and they had more comorbidities as well as higher ECOG and ASA scores (American Society of Anesthesiologists) (Table 1). Since higher age and comorbidities (e.g. dementia, liver disease and anaemia) have been described as negative predictive factors for PFS and OS [32–35], our data demonstrate improved oncological efficacy of IO/TKI treatment combinations of even in older, less fit and health-impaired patients. However, there are also studies showing that age alone has no effect on PFS and OS in this setting [36–38]. Further analysis of comorbidities and patient characteristics is needed to clarify their influence on the oncological efficacy of systemic therapies in patients with aRCC.

Contrary to clinical intuition, the overall incidence of TRAEs was balanced between the two treatment groups (Table 3). Even higher-grade side effects (grade 2 and 3 according to CTCAE) were balanced between the two groups (Table 3). However, life-threatening adverse events with a CTCAE grade of 4 were only observed in the IO/TKI group (Table 3). As a result, there was a significant increase in the number of interdisciplinary consultations for the management of adverse events and more frequent TRDD (Table 3). This effect is most likely due to the use of TKIs. In 5 of the 8 patients, a life-threatening cardiovascular event (2 × apoplex, 1 × pulmonary embolism, 1 × subarachnoid haemorrhage and 1 × atrioventricular block (grade 3) with resuscitation and insertion of a pacemaker) occurred during treatment with IO/TKIs. This observation is probably due to the known cardiovascular side effect profile of TKIs [39–42]. In addition, TRAEs with TKIs are known to occur particularly in older patients [36]. As the patients in the IO/TKI group were noticeably older and more health-impaired, this observation could be explained by the poorer health configuration of the patients. The high number of life-threatening TRAEs also explains the noticeably higher number of TRDDs (Table 3), as the continuation of medication is no longer recommended for serious TRAEs [43]. Due to the possible gradual dose reduction of TKIs, a dose reduction was of course only seen in this group. However, this was necessary in > 50% of the patients (Table 3), which could also be due to the poorer overall health constitution. Interestingly, the use of glucocorticoids was higher in the IO/IO group (Table 3). The possibility of dose reduction may have resulted in patients in the IO/TKI group needing less glucocorticoids. However, it is clear that good management of side effects and close monitoring of patients undergoing immunotherapy have a significant impact on the success of treatment and therefore require a high level of interdisciplinary expertise from the treatment team [20, 21, 44–46].

In summary, our study provides important real-world data on the comparative efficacy of current first-line therapies and the influence of patient characteristics, comorbidities, side effects and treatment management in aRCC. We were able to show that an IO/TKI combination significantly improves PFS compared to IO/IO, which is in line with recent study results. In terms of OS, we did not see a significant improvement. However, close monitoring and adequate side effect management should be ensured, especially in older and more health-impaired patients. We are contributing to the still sparse data on the efficacy of these two key treatment regimens, as the choice of first-line treatment for aRCC remains uncertain.

Our study also has some limitations. Due to the monocentric study design, the study population is relatively small, resulting in a rather heterogeneous overall study population. There were also large differences in the distribution of patients between the different combination therapies. Therefore, subgroup analysis or matched-pair analysis was not possible due to low statistical power. This also makes it difficult to compare the effectiveness of the individual combination therapies between each other and to analyse important secondary endpoints, such as duration of response. In addition, the observation period of the patient population was not long enough to evaluate long term side effects.

## Conclusion

In the real-world setting, IO/TKI combination therapy appears to be associated with improved PFS and ORR compared to IO/IO for first-line treatment of aRCC, even in older and more health-impaired patients. However, careful monitoring and interdisciplinary management remain crucial, as this patient group may be at higher risk for severe side effects. Additionally, synchronous metastases were identified as a key risk factor for both PFS and OS. Further research is needed to confirm whether the observed oncological benefits of IO/TKI are sustained over time and to provide stronger evidence for a more individualized treatment approach based on patient-specific factors.

## Supplementary Information

Supplementary material 1.

## Data Availability

Research data are not publicly available on legal and ethical grounds to protect the privacy of research participants. Research data can be provided on request in pseudonymized form. Further enquiries can be directed to the corresponding author.
