# Supplementary material for: Real-world comparison of the efficacy of first-line therapies and the influence of risk factors in advanced renal cell carcinoma
Source: Discov Oncol. 2025 Mar 19;16:359. doi: 10.1007/s12672-025-02131-z (PMC11923313; doi:10.1007/s12672-025-02131-z)
Supplement: Supplementary file 1 — Supplementary material 1. [file 12672_2025_2131_MOESM1_ESM.docx]

**SUPPLEMENTARY MATERIAL**

**Supplementary Table 1:** Univariate Cox regression analysis for progression free survival (PFS). Depicted are hazard ratio, lower and upper confidence interval (CI). P<0,05 was considered significant.

| **Predictor for PFS** | **Hazard Ratio** | **lower CI** | **upper CI** | **p-value** |
| --- | --- | --- | --- | --- |
| **Comorbidities**  History of smoking  Arterial hypertension  Atrial fibrillation  Coronary artery disease  Anemia  Liver cirrhosis  Diabetes mellitus  Chronic kidney disease  COPD  2^nd^ cancerous disease  Thyroid disease  Colitis  Neurologic disorder  **Distribution of metastasis**  pulmonary  visceral  cerebral  osseous  lymphoid  **Synchronous metastasis**  Use of Corticosteroids  **Treatment with IO/TKI** | 1.15  1.06  1.11  0.68  0.73  0.97  0.65  0.83  0.36  0.51  0.20  1.77  1.70  0.53  1.06  0.21  1.43  1.49  **2.42**  0.84  **0.46** | 0.60  0.55  0.46  0.15  0.26  0.28  0.27  0.32  0.11  0.20  0.03  0.24  0.68  0.27  0.56  0.03  0.71  0.67  **1.13**  0.39  **0.23** | 2.21  2.03  2.66  3.04  2.11  3.34  1.56  2.15  1.18  1.31  1.46  13.08  4.22  1.04  2.03  1.55  2.86  3.29  **5.15**  1.79  **0.90** | 0.681  0.865  0.824  0.616  0.565  0.963  0.333  0.695  0.090  0.162  0.112  0.576  0.253  0.063  0.851  0.126  0.315  0.328  **0.023**  0.640  **0.023** |

**Supplementary Table 2:** Univariate Cox regression analysis for overall survival (OS). Depicted are hazard ratio, lower and upper confidence interval (CI). P<0,05 was considered significant.

| **Predictor for OS** | **Hazard Ratio** | **lower CI** | **upper CI** | **p-value** |
| --- | --- | --- | --- | --- |
| **Comorbidities**  History of smoking  Arterial hypertension  Atrial fibrillation  Coronary artery disease  Anemia  Liver cirrhosis  Diabetes mellitus  Chronic kidney disease  COPD  2^nd^ cancerous disease  Thyroid disease  Colitis  Neurologic disorder  **Distribution of metastasis**  **pulmonary**  visceral  cerebral  osseous  lymphoid  **Synchronous metastasis**  Use of Corticosteroids  Treatment with IO/TKI | 1.21  0.97  1.49  0.97  0.26  0.40  0.69  0.69  0.36  0.81  0.25  1.57  1.11  **0.42**  1.26  0  1.25  1.75  **3.79**  0.53  0.64 | 0.54  0.42  0.54  0.20  0.03  0.05  0.23  0.20  0.11  0.30  0.03  0.21  0.36  **0.18**  0.56  0  0.51  0.57  **1.28**  0.18  0.27 | 2.73  2.26  4.05  4.62  1.98  3.27  2.03  2.39  1.18  2.20  1.91  11.87  3.39  **0.97**  2.81  9.99E+999  3.08  5.37  **11.27**  1.59  1.51 | 0.641  0.948  0.440  0.967  0.193  0.394  0.494  0.561  0.083  0.680  0.180  0.660  0.854  **0.043**  0.579  0.994  0.624  0.330  **0.016**  0.258  0.307 |

**Supplementary Table 3:** Multivariate Cox regression analysis for progression free survival (PFS). Depicted are hazard ratio, lower and upper confidence interval (CI). P<0,05 was considered significant.

| **Predictor for PFS** | **Hazard Ratio** | **lower CI** | **upper CI** | **p-value** |
| --- | --- | --- | --- | --- |
| ***All IMDC groups***  **Synchronous metastasis**  **Treatment with IO/TKI**  ***Only intermediate/poor***  **Synchronous metastasis**  **Treatment with IO/TKI** | **2.38**  **0.47**  **2.93**  **0.44** | **1.11**  **0.24**  **1.10**  **0.22** | **5.11**  **0.92**  **7.76**  **0.89** | **0.026**  **0.029**  **0.031**  **0.022** |

**Supplementary Table 4:** Multivariate Cox regression analysis for overall survival (OS). Depicted are hazard ratio, lower and upper confidence interval (CI). P<0,05 was considered significant.

| **Predictor for OS** | **Hazard Ratio** | **lower CI** | **upper CI** | **p-value** |
| --- | --- | --- | --- | --- |
| ***All IMDC groups***  **Synchronous metastasis**  Pulmonal metastasis  Treatment with IO/TKI  ***Only intermediate/poor***  **Synchronous metastasis**  Treatment with IO/TKI | **3.47**  0.47  0.62  **3.73**  1.53 | **1.15**  0.20  0.26  **1.06**  0.63 | **10.44**  1.10  1.49  **13.11**  3.72 | **0.027**  0.082  0.283  **0.040**  0.347 |


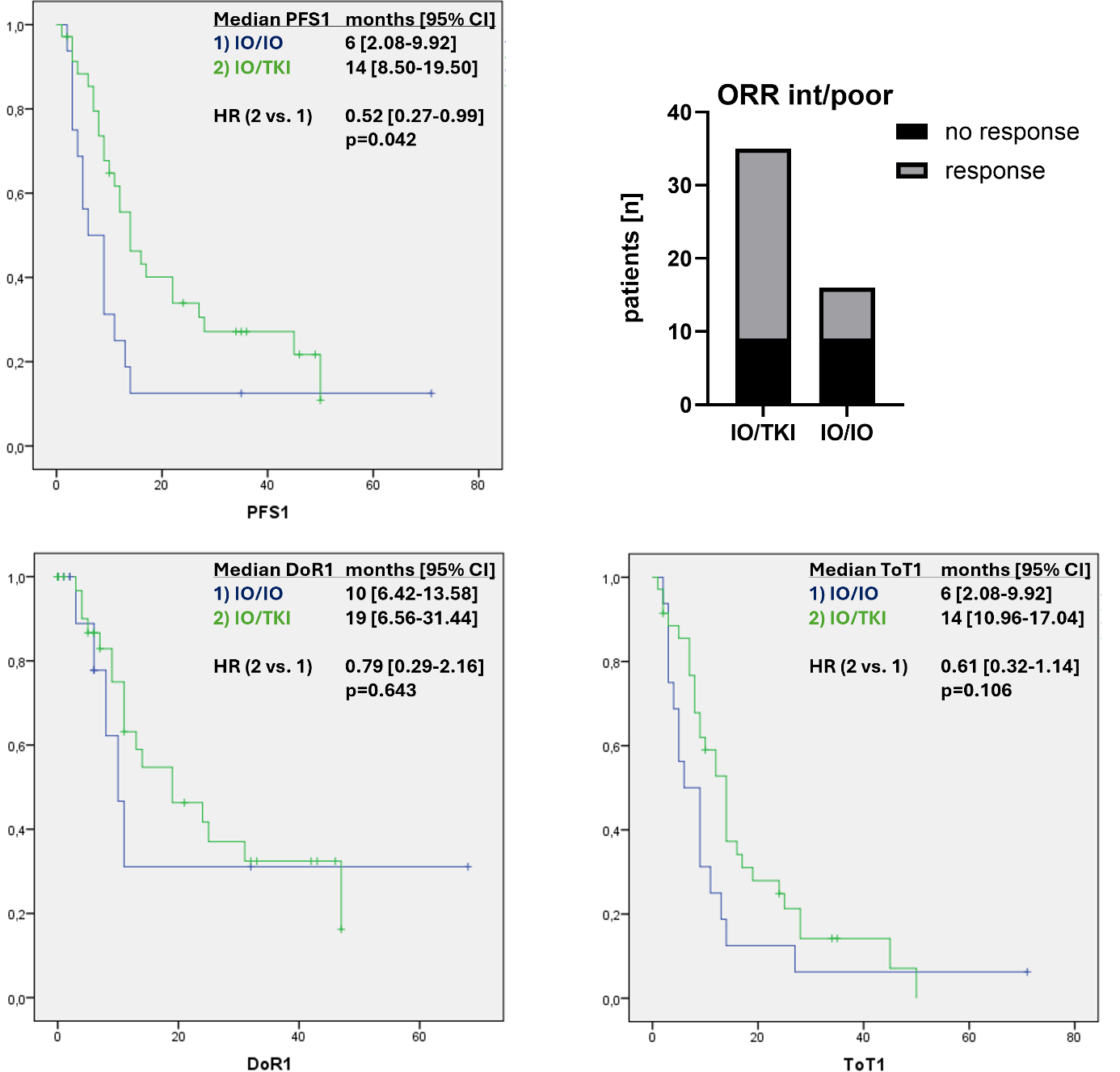


**Supplementary Figure 1:** Kaplan-Maier analysis of progression-free survival (PFS1), duration of response (DoR1), time on treatment (ToT1) as well as objective response rate (ORR) of the first line treatment compared between the two therapy regimes (1. IO/IO and 2. IO/TKI) only in patients with intermediate and poor risk prognosis (after IMDC). Peto-Pike’s Chi^2^ was used for Log-Rank-Test. Hazard ratios (HR) with 95 % confidence intervals (CI) were approximated via Peto-Pike. P<0,05 was considered significant.


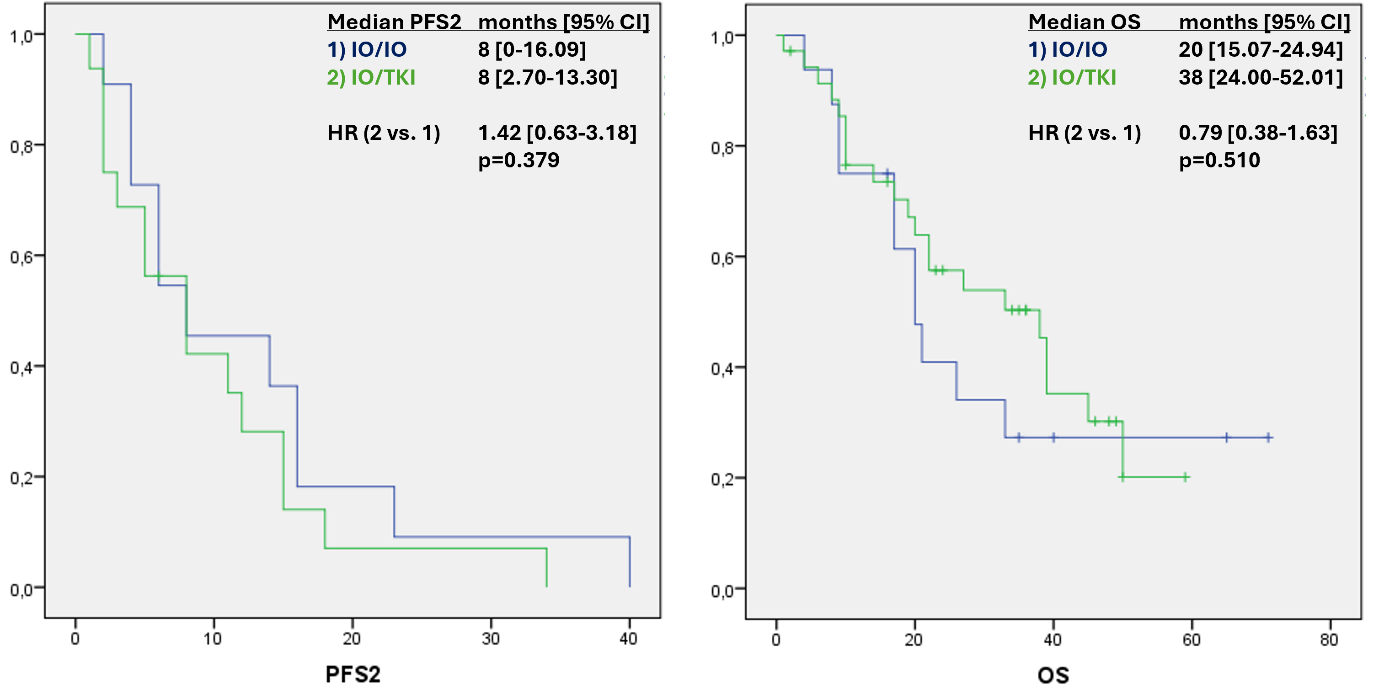


**Supplementary Figure 2:** Kaplan-Maier analysis of progressive free survival of second line treatment (PFS2) and overall survival (OS) compared between the two therapy regimes (1. IO/IO and 2. IO/TKI) only in patients with intermediate and poor risk prognosis (after IMDC). Peto-Pike’s Chi^2^ was used for Log-Rank-Test. Hazard ratios (HR) with 95 % confidence intervals (CI) were approximated via Peto-Pike. P<0,05 was considered significant.


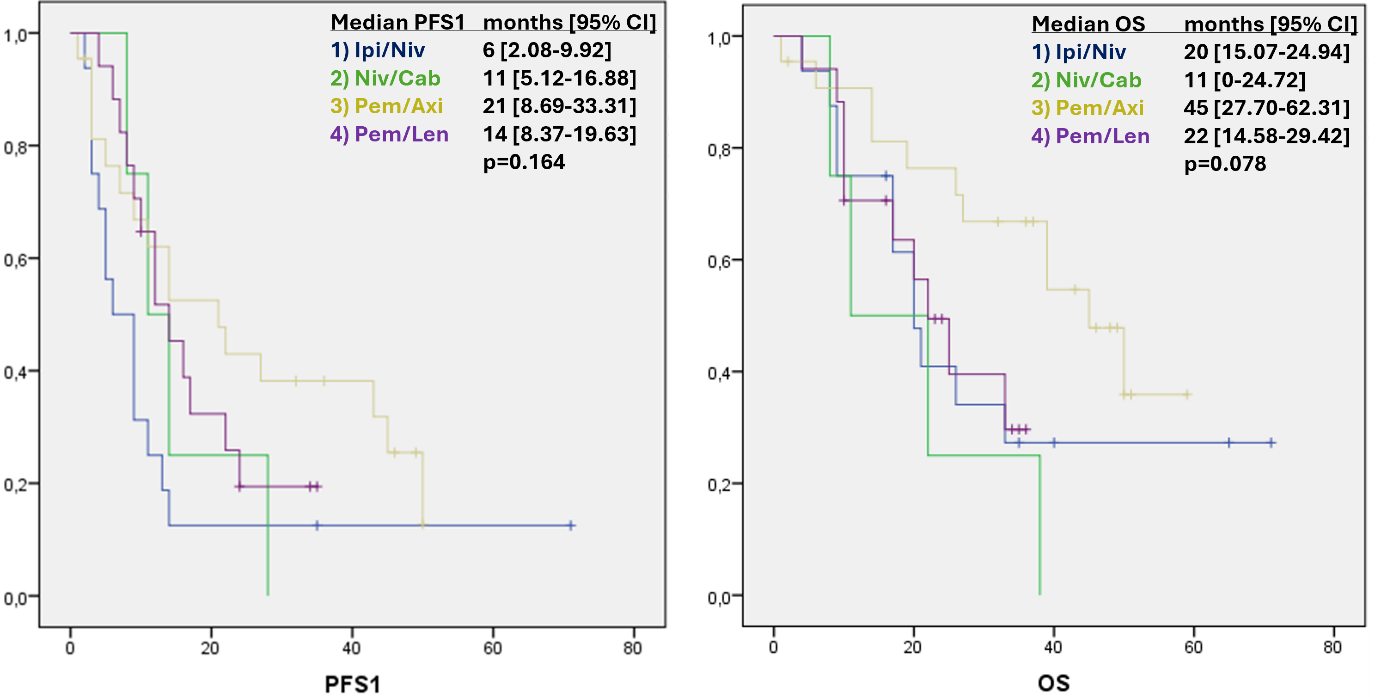


**Supplementary Figure 3:** Kaplan-Maier analysis of progressive free survival of first line treatment (PFS1) and overall survival (OS) compared between the individual treatment combinations. Peto-Pike’s Chi^2^ was used for Log-Rank-Test. Hazard ratios (HR) with 95 % confidence intervals (CI) were approximated via Peto-Pike. P<0,05 was considered significant.


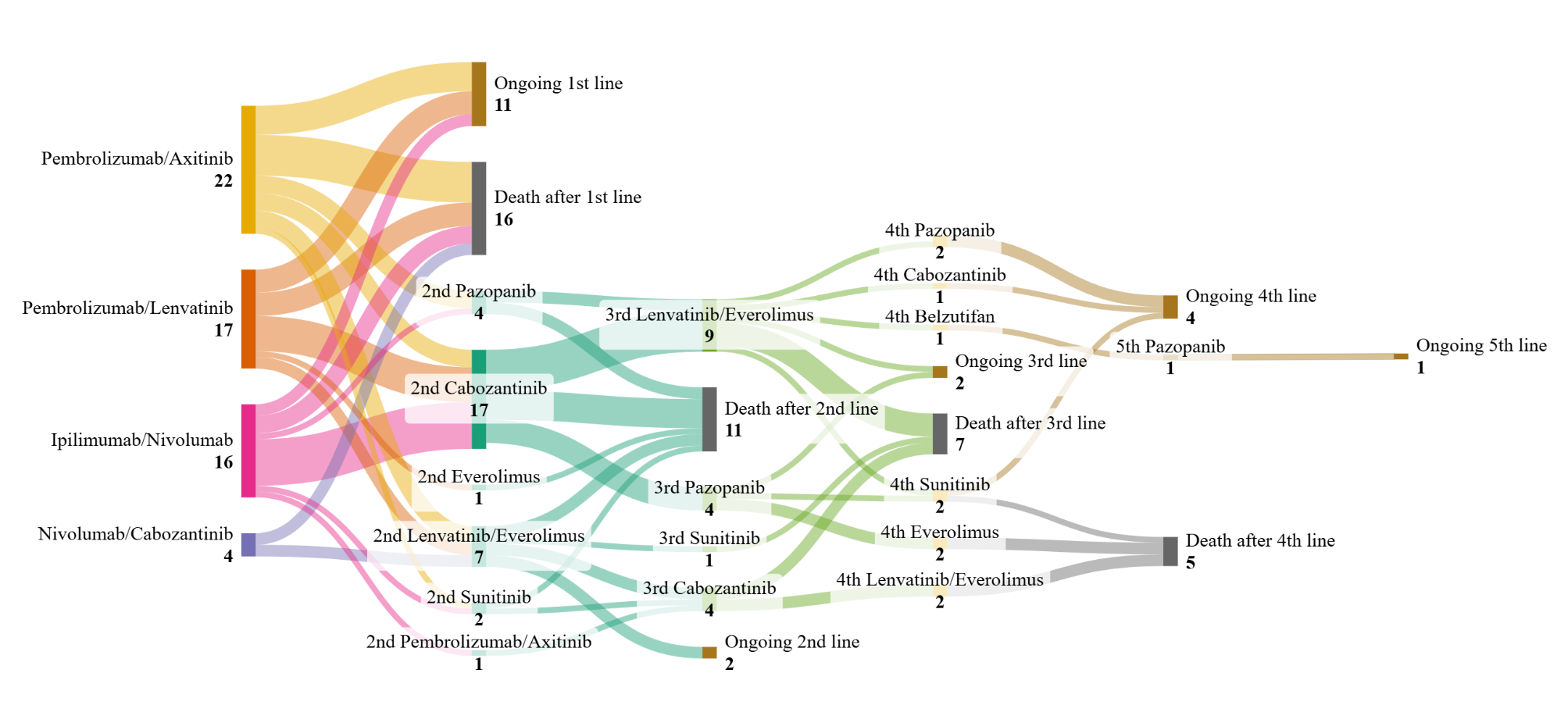


**Supplementary Figure 4:** Sankey diagram of all treatment lines of the entire patient population with the corresponding number of patients in each treatment line.

**Supplementary Figure 5:** Median follow-up in months between the two treatment groups.
